# The undiscovered syndrome: Macdonald Critchley’s case of semantic dementia

**DOI:** 10.1080/13554794.2014.910307

**Published:** 2014-05-12

**Authors:** Pirada Witoonpanich, Sebastian J. Crutch, Jason D. Warren, Martin N. Rossor

**Affiliations:** ^a^ Dementia Research Centre, UCL Institute of Neurology, University College London, London, UK

**Keywords:** semantic dementia, Macdonald Critchley, historical, nosology

## Abstract

Semantic dementia is a unique clinicopathological syndrome in the frontotemporal lobar degeneration spectrum. It is characterized by progressive and relatively selective impairment of semantic memory, associated with asymmetric antero-inferior temporal lobe atrophy. Although the syndrome became widely recognized only in the 1980s, descriptions of cases with typical features of semantic dementia have been on record for over a century. Here, we draw attention to a well documented historical case of a patient with features that would have fulfilled current consensus criteria for semantic dementia, as reconstructed from the notes made by her neurologist, Macdonald Critchley, in 1938. This case raises a number of issues concerning the nosology of the semantic dementia syndrome and the potential value of archived case material.

Semantic dementia (SD) is a unique clinicopathological syndrome in the frontotemporal lobar degeneration (FTLD) spectrum. It is characterized by progressive and relatively selective impairment of semantic memory, the memory system mediating knowledge of words, objects, and concepts based on our prior experience of the world (Hodges & Patterson, 2007). SD is associated with asymmetric antero-inferior temporal lobe atrophy and abnormal cellular deposition of a particular pathogenic protein (TDP-43) in the majority of cases (Fletcher & Warren, 2011; Hodges & Patterson, 2007).

The clinical syndrome of SD was described first by Arnold Pick in 1892 (Pick, 1892; see Spatt, 2003). Pick identified a syndrome of progressive “amnesic aphasia” with loss of memory for words in the context of focal cerebral atrophy (Pick, 1931). However, modern interest in the SD syndrome awaited the work of Warrington (Warrington, 1975) who described three patients with cerebral atrophy and impaired knowledge about verbal and pictorial representations of objects. Her key insight was to recognize that these patients’ agnosia for words and visual objects represented two halves of a coherent syndrome, a primary disturbance of the semantic memory system. Further attention was drawn to the syndrome by Mesulam (Mesulam, 1982) who highlighted the existence of a wider spectrum of progressive language disorders in the absence of generalized dementia, subsequently termed “primary progressive aphasia” (PPA) (Mesulam, 1987). The term “semantic dementia” was coined by Snowden and colleagues (Snowden, Goulding, & Neary, 1989) to describe three patients with fluent, empty speech, and impaired naming and single word comprehension; this label captured the progressive nature of the language and the semantic memory impairment. Subsequently, Hodges and colleagues (Hodges, Patterson, Oxbury, & Funnell, 1992) linked SD with a characteristic anatomical pattern of bilateral but generally asymmetric anterior temporal lobe atrophy and one of Warrington’s original three patients was subsequently shown to have characteristic pathology (Rossor, Revesz, Lantos, & Warrington, 2000). Diagnostic criteria for SD (Gorno-Tempini et al., 2011; Neary et al., 1998) have been proposed to fit the syndrome in the FTLD spectrum; these criteria have underlined the status of SD as one of the three prototypical syndromes of FTLD and PPA, reflected in an explosion of clinical and research interest in the condition (Hodges & Patterson, 2007; Lambon Ralph, Sage, Jones, & Mayberry, 2010).

Macdonald Critchley (1900–1997) was a neurological polymath and one of the founding fathers of modern cognitive and behavioral neurology (Compston, 2010; Martinez, Moro, Munhoz, & Teive, 2013), authoring such classic texts as The Parietal Lobes and Music and the Brain. He was active chiefly at the National Hospital for Neurology and Neurosurgery (NHNN) in London, where his clinical service spanned some six decades and where he was renowned as an astute observer of neurological disease, particularly in the realm of cognitive and language disorders. The NHNN and the Institute of Neurology, University College London, jointly maintain archived case dating from the time of the founding of the Hospital in 1859 (Queen Square Archives, http://www.queensquare.org.uk/archives/), and collectively these constitute an invaluable historical and clinical resource. Access to the archives has proved crucial in characterizing rare entities such as familial British dementia (Mead et al., 2000) and continues to provide an unparalleled testament to the development of British neurology and modern clinical neurological thought.

We recently had the opportunity to review from the Archives the case records of a patient under the care of Macdonald Critchley in 1938, whose clinical description was in striking accord with the SD syndrome as it is presently understood. We now put this historical case on record, and consider the implications raised by such cases for the nosology of SD and neurological disease, and the value of archived clinical case material.

## Case description

**Figure 1.**
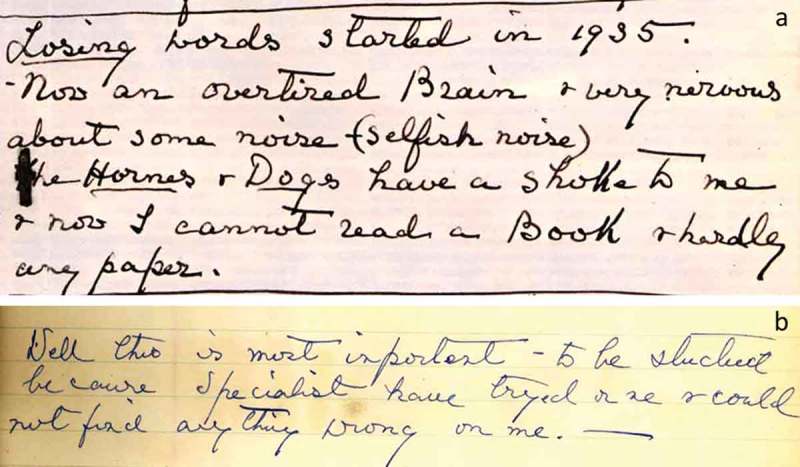
The patient’s writing describing her symptoms (a). Prominent errors on spelling and preposition (b). She made mistakes as following; “importent” for “important”, “specialist” for “specialists”, “tryed” for “tried”, “onse” for “once” and “on me” for “with me”. [To view this figure in color, please see the online version of this Journal.]

This 51 year old right-handed English widow was admitted with progressive dysphasia to the National Hospital under the care of Dr Critchley (clerked by Dr Paul Sandifer) in October 1938. She presented with progressive disturbance of language unfolding over four years (Figure 1a). Initially, this chiefly affected her ability to find the names of things; however, by the time of her admission she was having difficulty also in understanding spoken or written words sufficient to interfere with her taking part in conversations or attending the theatre. She said, “I often recognize words but I haven’t the faintest idea what they mean. It’s almost as if they were foreign”. Her reading had become “most laborious and she never ‘read’ anything now as she comprehends only a proportion of the words she sees” and her spelling had also deteriorated. Her episodic memory (for “events and experiences”) and arithmetical skills were unaffected. She was also noted over the past 10 years to have become nervous, self-centered, and argumentative. She had taken to attending séances in an attempt to communicate with her deceased husband, and believed that she was able to do so. She remained able to appreciate music and painting and felt that her sense of humor had grown “keener”. However, she complained bitterly about ambient noises, “selfish noises like the sharp blare of motor cars or barking dogs”; she found these common everyday sounds intensely irritating and distracting. Samples of the patient’s written discourse contain repeated references to this increased sensitivity to sounds (Figure 1b). Although she remained able to play the piano competently, she had difficulty simultaneously coordinating the treble and bass parts. There was a suggestion that she retained familiarity with some songs she could no longer name and was able to sing but not speak or understand their lyrics.

There was no significant past medical history or family history of any mental or nervous disease. Both her parents had lived to an advanced age.

**Figure 2.**
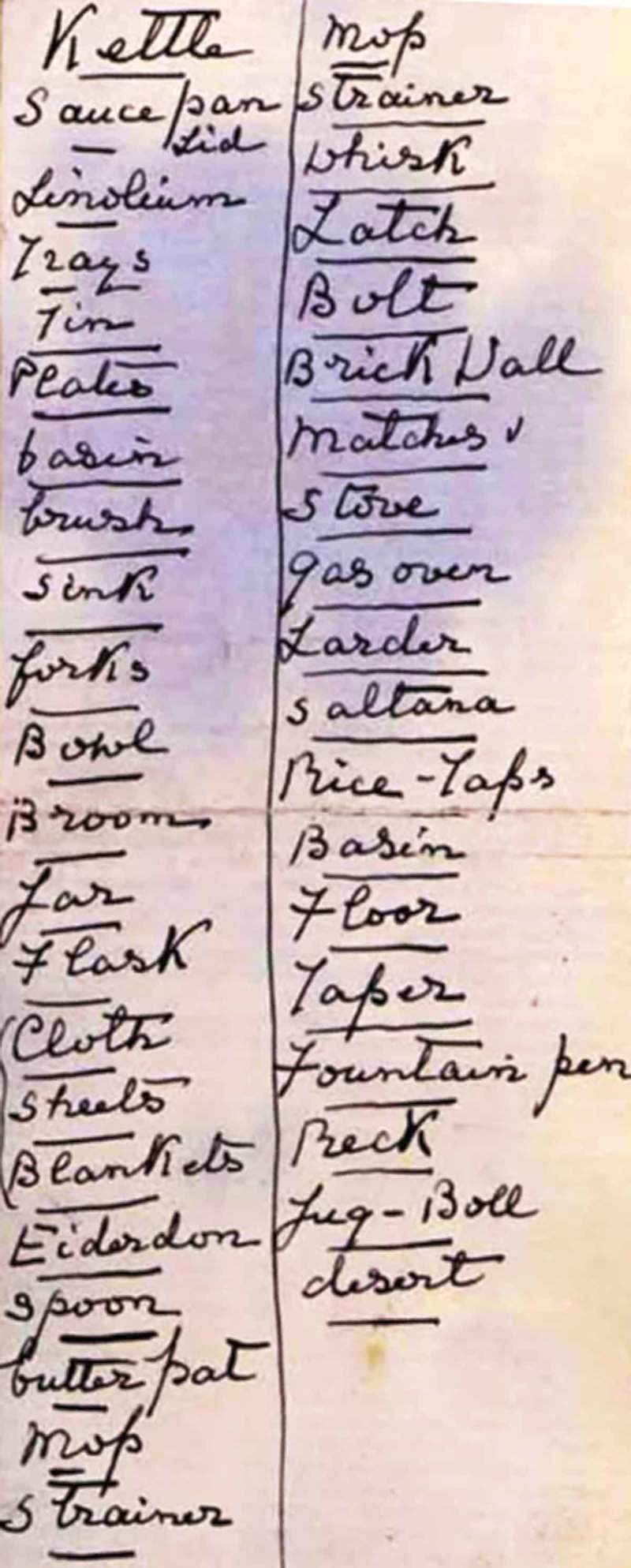
Lists of words whose meanings the patient no longer understood (compiled with the help of her friend). [To view this figure in color, please see the online version of this Journal.]

**Figure 3.**
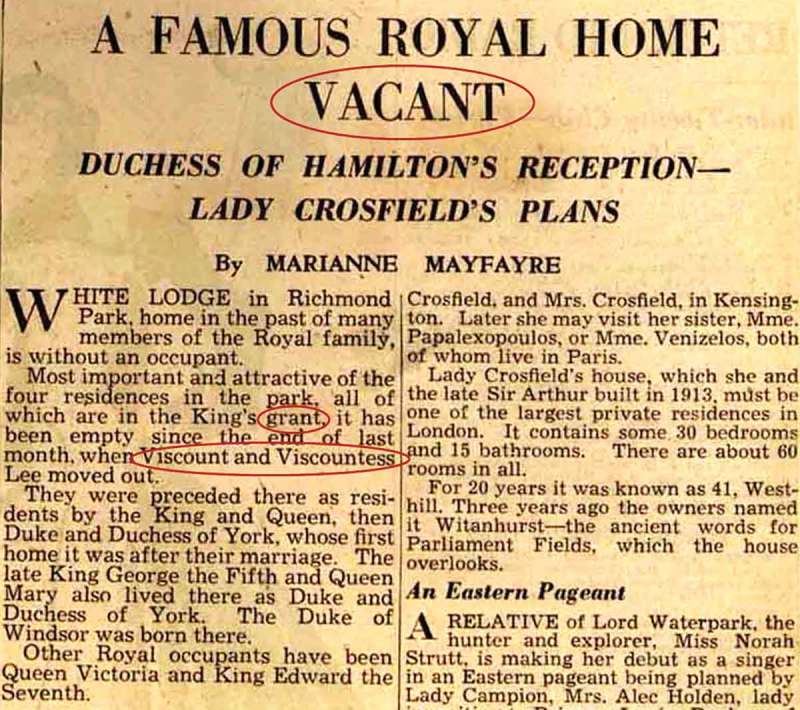
Newspaper passage for reading aloud. Words on which the patient made regularization errors are circled in red. [To view this figure in color, please see the online version of this Journal.]

**Figure 4.**
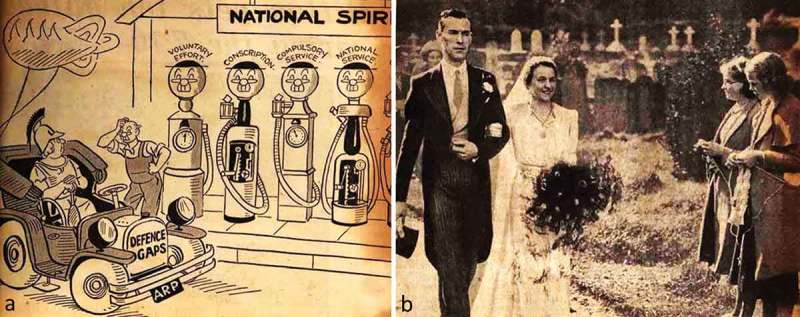
Pictures for assessing visual object recognition. See text. [To view this figure in color, please see the online version of this Journal.]

When examined, she was noted to have an unusual personality, and to be distractible and garrulous. It was commented that “on hearing her prattle, nothing strikingly abnormal was at first noticed but mistakes were detected not expected in her stratum of society”. Examples included “recovered me” for “cured me” and “I am very physical” for “I am very healthy”. Although her speech was well articulated and grammatical, she was noted to make frequent use of circumlocutions and to have particular difficulty finding names. The patient was unable to name a pencil, torch, tape measure, or towel. There was further evidence of a “gross agnosia” for words. She brought with her extensive lists of words whose meanings she no longer knew, prepared with the assistance of a friend (Figure 2); examples include “kettle”, “saucepan”, “floor”, “fountain”, and “desert”. She was able to repeat words normally. Reading aloud from a newspaper clipping (Figure 3) was slow and labored and marred by regularization errors, for example, sounding the first vowel in “vacant” as short and sounding the “s” in “Viscount”. She made similar errors when writing (Figure 1b), for example, rendering “tried” as “tryed” and “once” as “onse”. A more detailed assessment of her “amnesia for words” revealed that this affected mainly nouns, including body parts (“nose”, “finger”) but also extended to adjectives (“narrow”) and adverbs (“quickly”). She was unable to match objects with their spoken names. Although she was able to manipulate everyday objects normally when these were presented to her, there was a suggestion of visual agnosia for pictures. When shown a cartoon including petrol pumps (Figure 4a), she interpreted these as clocks and failed to recognize a balloon in the shape of an airship; she misinterpreted a wedding photograph (Figure 4b) as a funeral on the strength of the churchyard tombstones in the scene. She was, however, still able to recognize everyday sounds and noises. She was able to draw a simple map of a locale in France where she had taken her vacations. The general neurological and systemic examinations were normal.

The patient underwent examination of the cerebrospinal fluid, which was unremarkable.

A diagnosis of organic dementia with dysphasia was made.

## Discussion

This patient exhibited many of the clinical features we would now regard as classical for the SD syndrome. She presented with progressive fluent aphasia manifesting as early severe anomia and loss of comprehension of words. She also demonstrated loss of vocabulary-based reading and spelling characterized by a surface dyslexia and dysgraphia but with intact grammar, phonology, and repetition. Indeed, these language features would have fulfilled current consensus diagnostic criteria for the “semantic variant” of PPA (Gorno-Tempini et al., 2011); in addition to this leading deficit of verbal semantic memory, there was evidence for semantic impairment affecting recognition of visual objects. Semantic impairment in SD, though ultimately panmodal (Goll et al., 2010; Omar, Mahoney, Buckley, & Warren, 2013), is most often led by verbal semantic impairment (Hodges & Patterson, 2007) with initially more variable involvement of other knowledge modalities. Also consistent with the focality of cognitive deficits in SD, the patient had sparing of a number of cognitive domains, including episodic (and probably also topographical) memory, calculation, and praxis. On the other hand, there was the suggestion of executive dysfunction, for example, when playing the piano (a task requiring coordination of simultaneous actions), and, more significantly, alterations in behavior and personality, including anxiety, irritability, hyper-religiosity, lack of empathy, mental rigidity, and preoccupations. These features are now recognized to be common accompaniments of SD (Rohrer & Warren, 2010), perhaps reflecting spread of the pathological process to involve orbitofrontal cortex. Another strikingly prominent behavioral feature in this case was hyperacusis, to which patients with SD have been shown to be predisposed (Mahoney et al., 2011). Although not documented in detail, the patient’s retained musical interest and competence would also be in keeping with recent reports of musicophilia and relatively preserved musical knowledge in SD (Fletcher, Downey, Witoonpanich, & Warren, 2013; Hailstone, Omar, & Warren, 2009)

Beyond its historical interest, this case raises a wider issue concerning the nosology of the SD syndrome. Arguably to a more striking degree than any other syndrome in cognitive neurology, SD illustrates the coherent conjunction of a clinical syndrome with a cognitive module (semantic memory failure) and an anatomical substrate (focal anterior temporal lobe atrophy) (Fletcher & Warren, 2011; Hodges & Patterson, 2007). Macdonald Critchley was among the most skilled neurological observers of the last century and had a highly sophisticated understanding of the human language system and its disorders informed by a wide-ranging acquaintance with the published literature in these disorders, as illustrated in his numerous publications including Aphasiology (Critchley, 1970a). However, Critchley may not have appreciated the true significance of the cognitive syndrome in this case. Indeed, in his later writings he inclined to the view that aphasia in dementia generally reflects a superadded disease process (Critchley, 1970b). Judging the past by contemporary standards is the classic historian’s fallacy; for the SD syndrome, however, a detailed clinical description had already been published by Arnold Pick (Pick, 1892) some years prior to the recording of this case. In Aphasia (Pick, 1931), Pick refers to progressive “amnesic aphasia” as a distinct syndrome characterized by loss of words corresponding to ideas with reliance on paraphrasis but an intact “lively” speech structure. Pick went on to state that “in severe cases, ‘aphasia’ is followed by inability to indicate the purpose of ‘an object’”, which probably refers to visual agnosia. He further recognized that “early involvement of the left temporal lobe in atrophic processes… probably plays the major role here”. Critchley’s patient was characterized as having “amnesic aphasia”; this case could, therefore, have been aligned with Pick’s neurodegenerative cases, and thereby might have stimulated interest in the SD syndrome decades before its eventual rediscovery.

The present example of SD echoes a number of instances in the history of neurology in which disorders were described clearly but their significance was not appreciated until later when the necessary nosological framework became available. Indeed, the very concept of functional localization in cerebral cortex was proposed by Swedenborg centuries before it became widely accepted (Gross, 1998). A further issue raised by this case, and a plausible reason why the link was not made with earlier descriptions of similar cases, is the problem of accessing relevant information. Pick published in German, and Aphasia (Pick, 1931), which contains perhaps his clearest and most succinct formulation of the progressive amnesic aphasia syndrome, was not translated into English until 1973; nor is this an isolated instance in the field (Rosenfeld, 2001; Snowden, 2001). It is possible that Critchley simply lacked access to pertinent case materials. The ongoing contemporary revolution in medical information technology has granted us unprecedented access to the accumulated clinical and scientific literature; and yet, this combinatorial explosion of resources may paradoxically allow potentially crucial information to go undetected. The most important and relevant legacy of historical cases such as Critchley’s and Pick’s may be to underline the value of archived material and to stimulate today’s information-overburdened neurologists to look beyond the English-speaking world when mining this historic legacy.
